# A Systematic Review of the Impact and Outcome of Loneliness and Self-Isolation on Individuals Living With Cardiovascular Disease

**DOI:** 10.7759/cureus.81277

**Published:** 2025-03-27

**Authors:** Collins C Okeke, Angela Ojo, Onyinye Ngige, Chukwunomso C Amuchie, Chibuike F Obi, Oyiyechukwu R Charles, Ifunanya R Ekeocha, Dumebi C Konyeme, Kris N Idion, Favour O Balogun, Ijeoma Onwuchekwa, Euodia A Ugo-Ihanetu, Ginika S Okafor, Davida E Ogona, Chidera C Okoye, Somadila A Igboanugo

**Affiliations:** 1 Anaesthesiology, Surgery Interest Group of Africa, Lagos, NGA; 2 Internal Medicine, University of Port Harcourt, Rivers State, NGA; 3 Internal Medicine, Afe Babalola University, Ado Ekiti, NGA; 4 Internal Medicine, Nnamdi Azikiwe University Teaching Hospital, Nnewi, NGA; 5 General Practice, Accra College of Medicine, Accra, GHA; 6 Acute Medicine, Fairfield General Hospital, Bury, GBR; 7 Research, Surgery Interest Group of Africa, Lagos, NGA; 8 Hematology, Delta State University Teaching Hospital, Oghara, NGA; 9 Paediatrics, Afe Babalola University, Ado Ekiti, NGA; 10 Internal Medicine, Delta State University Teaching Hospital, Oghara, NGA; 11 Internal Medicine, James Paget University Hospital, Great Yarmouth, GBR; 12 Oncology, Royal Free Hospital, London, GBR; 13 Internal Medicine, Rivers State University Teaching Hospital, Port Harcourt, NGA; 14 Internal Medicine, Federal Teaching Hospital Ido-Ekiti, Ido-Ekiti, NGA; 15 Cardiology, Asaba Specialist Hospital, Okpanam, NGA; 16 Internal Medicine, Asaba Specialist Hospital, Okpanam, NGA; 17 Cardiology, Rostov State Medical University, Rostov-on-Don, RUS

**Keywords:** cardiovascular disease, impact, loneliness, outcome, self-isolation, cardiovascular diseases

## Abstract

Social isolation and loneliness seriously impact the physical and mental quality of health and longevity. Social isolation and loneliness are important yet neglected social determinants for people of all ages. Cardiovascular diseases (CVD) are among the leading causes of death globally, and loneliness has been linked to worsening outcomes of CVD. This review aims to evaluate the impact of loneliness/social/self-isolation among adults living with any cardiovascular disease.

A systematic search was done from inception to 23rd January 2025 on PubMed and Google Scholar, and 4,198 articles were synthesized; these articles were screened for duplication, and the duplicate articles were removed. A total of 3,606 articles underwent title and abstract screening, and 3,505 articles were removed following the predefined eligibility criteria. One hundred and one articles underwent full-text screening, and only 13 articles were included for qualitative analysis. We included adults aged 18 and above living alone/socially isolated with any cardiovascular disease.

This systematic review analyzed 13 studies across eight countries, encompassing 63,978 participants, of whom 12,110 were identified as living alone or socially isolated, 7,264 male and 3,745 female participants; one study included only male participants, while two studies didn't mention the number of male and female participants. The findings provide compelling evidence that loneliness is associated with adverse cardiovascular disease (CVD) outcomes, including increased mortality, recurrent cardiovascular events, and higher hospitalization rates.

Addressing loneliness and social isolation should be a priority in cardiovascular disease prevention and management. Integrating social interventions could significantly improve the prognosis of individuals living with CVD, ultimately reducing morbidity and mortality rates in this vulnerable population.

## Introduction and background

High-quality social connections are essential to our mental and physical health and well-being. Social isolation and loneliness seriously impact the physical and mental quality of health and longevity. Social isolation and loneliness are important yet neglected social determinants for people of all ages, especially among the elderly [1]. Loneliness can be described as a subjective feeling of being alone, separated, or apart from others and has been conceptualized as an imbalance between desired and actual social contacts [2]. Social isolation can be described as the lack of social contact and having few people to interact with regularly. One can feel lonely while being with others [3]. Self-isolation is when an individual chooses to isolate/exclude/separate themselves from others for personal reasons. Veazie S, et al. defined social isolation as a state in which an individual lacks a sense of belonging socially, lacks an engagement with others, has a minimal number of social contacts, and is deficient in fulfilling a quality relationship [2]. Cardiovascular diseases (CVD) are the leading cause of death globally and comprise a group of disorders of the heart and blood vessels that include coronary heart disease, cerebrovascular disease, rheumatic heart disease, etc. Greater than four out of five CVD deaths are due to heart attacks and stroke [4]. Globally, about 17.9 million people die of CVD every year, according to the WHO [4]. In Europe, CVD represents the leading cause of noncommunicable deaths (∼50% of all deaths) [5]. Social isolation and loneliness are common, and it is estimated that 1 in 4 older people experience social isolation, and about 5-15% among adolescents [1]. According to the WHO, 20-34% of older people in China, India, the United States, and the regions of Europe and Latin America are lonely, and loneliness imposes a heavy financial burden on society [6]. A study has described Gen Z (adults between 18 and 22 years) as the loneliest generation, with factors attributed to higher social media use and less engagement in meaningful in-person activities. Also, the incidence of social isolation and loneliness increased after the COVID-19 pandemic [7].

Studies have shown a link between social isolation, loneliness, and death from CVD with a 29% increase in the risk of heart attack and a 32% increased risk of stroke and stroke mortality. Loneliness and social isolation are associated with a poor prognosis in individuals with existing coronary artery diseases or stroke [7].

Loneliness and self-isolation are uncommon in younger adults between 16 and 29 years old; however, these experiences may be a contributing factor to loneliness, bullying, moving to school, illness or disability, change in family circumstances, not getting on with family, not having money or taking part in social activities, social media, and isolation from a pandemic. However, risk factors for loneliness increased tremendously in the elderly, and they include facing bereavement, living alone, living with disability or illness, low fixed income, digital exclusion, caring for a partner, and reduced morbidity and loss of access to an affordable and/or suitable mode of transport [8]. The following risk factors have been identified to cause CVD: high blood pressure, physical inactivity, smoking, high cholesterol, obesity, and diabetes [9].

Several mechanisms have been hypothesized to link loneliness and poor cardiovascular outcomes, and they include increased stress reactivity, autonomic dysregulation, and exaggerated inflammatory response, which have been implicated in this pathway. Lonely individuals have been observed to have a higher total peripheral resistance in response to psychological stressors, increased resting systolic blood pressure, a low cardiac output, and lower heart rate variability in response to stress when compared with nonlonely individuals [10].

This review aims to evaluate the impact and outcomes of loneliness/social/self-isolation among adults living with any cardiovascular disease.

## Review

Methods

This systematic review was conducted according to the Preferred Reporting Items for Systematic Reviews and Meta-Analysis extension for systematic review (PRISMA), and the study protocol was registered with PROSPERO CRD420250648800.

Inclusion Criteria

The original article was written in English and published in a peer-reviewed journal from inception to January 2025. It reported the impact/effect and outcome of loneliness or self-isolation on adults (18 years and above) of any gender living with any cardiovascular disease.

Exclusion Criteria

For patients under the age of 18 who do not live with any cardiovascular disease or loneliness, articles published in other languages, animal studies, and study designs such as audits, opinions, conferences, reviews, case reports, meta-analyses, comments, and editorials were excluded.

A search was conducted on the 23rd of January 2025 on PubMed and Google Scholar using the following search phrases: (self-isolation) AND (cardiovascular diseases), (loneliness) AND (cardiovascular diseases), "loneliness," "cardiovascular disease," "self-isolation," and "cardiovascular diseases". The Google Scholar database remitted us access to the entire searched article, as we cannot go beyond page 49; hence, we were able to screen 980 articles.

Article duplicates, titles and abstract screening were conducted by four independent reviewers (C.C.O., A.O., C.F.O., and O.R.C.) against the predefined eligibility criteria, and Rayyan referencing manager [11] was used. After the abstract screening, eligible articles were subjected to full-text screening. Disagreements were discussed among reviewers, and another reviewer was invited in case no resolution was reached.

We extracted data from eligible articles, which include author name, study year, study design, sample size, mean age, individual with loneliness, type of card CVD, impact/effect, outcome, and follow-up. The included articles underwent quality assessment using the Joanna Briggs Institute (JBI) risk of bias critical appraisal tool for cohort, cross-sectional, and randomized control trials [12].

Results

**Figure 1:**
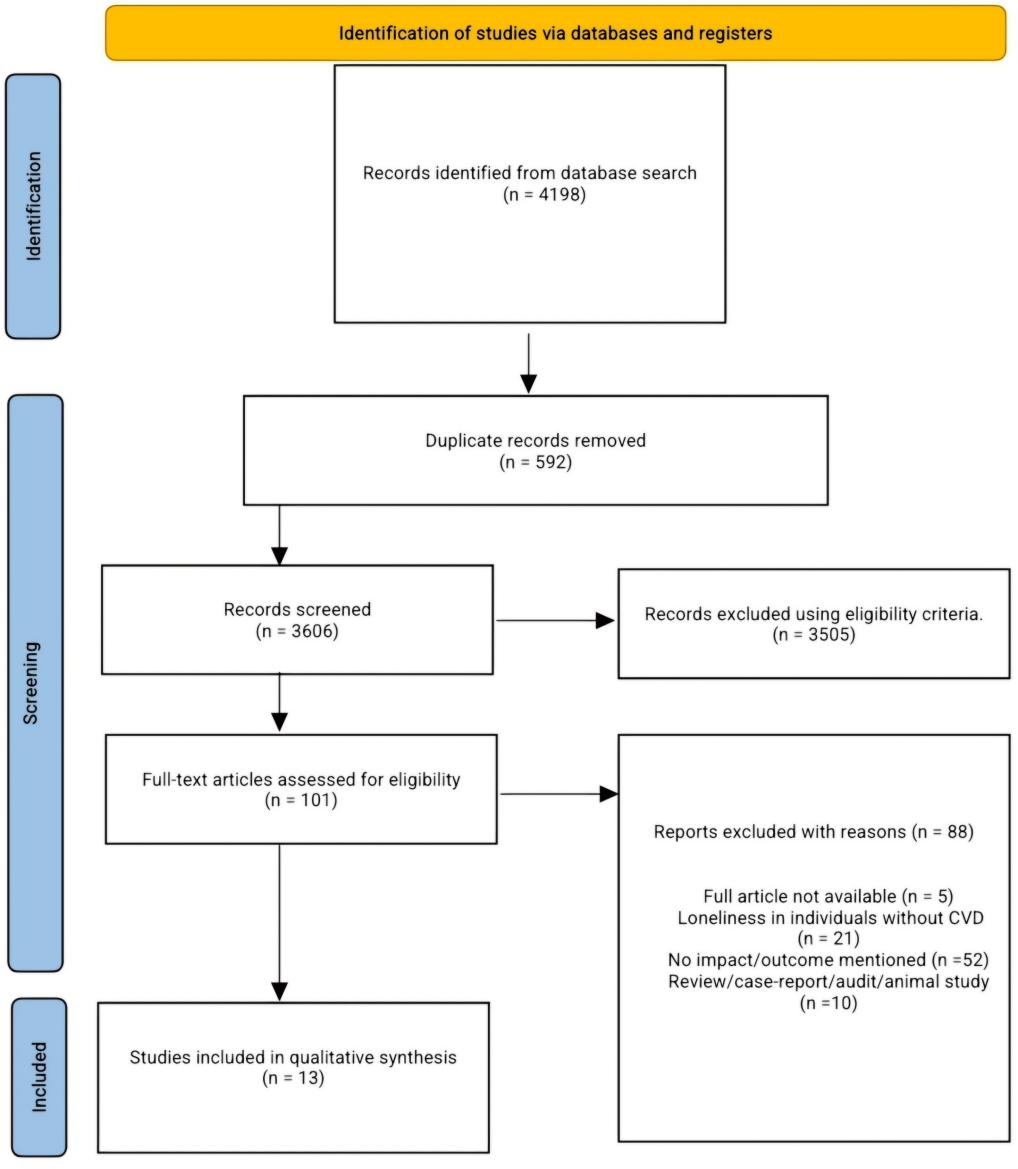
PRISMA flow diagram

Our systematic database search returned 4,198 articles; these articles were screened for duplication, and duplicated articles were removed. A total of 3,606 articles underwent title and abstract screening, and 3,505 articles were removed following the predefined eligibility criteria. One hundred and one articles underwent full-text screening, and only 13 articles were included for qualitative analysis and data extraction, while 88 articles were excluded due to the unavailability of full articles, identified individuals living with loneliness but not having any CVD, articles that didn't mention the impact/effect or outcome of loneliness among individuals living with CVD, and case reports, audits, reviews, and animal studies as shown in Figure 1.

Study Characteristics

A total of 63,978 participants from 13 articles from eight countries (Japan, the USA, Canada, Sweden, Taiwan, Finland, Spain, and Denmark) were included in this study. A total of 12,110 individuals were identified as living alone/socially isolated. The study period ranged from 1992 to 2024, the mean age ranged from 51.8 to 81 years, and the follow-up duration ranged from 362 days to 23 years. This study included 7,264 males and 3,745 females identified as living alone who participated in this study. Japan contributed three articles; the USA, Canada, and Sweden each contributed two articles, while Taiwan, Finland, Spain, and Denmark contributed one article each. Details of the study characteristics are shown in Table 1.

**Table 1:** Study characteristics

Author	Year	Country	Sample size	Living alone	Mean age	Male	Female	Follow up
Ohama et al. [13]	2017	Japan	208	45	68.9	34	11	3 years
Brummett et al. [14]	2000	USA	400	51	61.7	36	15	6 years
Gandhi et al. [15]	2010	Canada	32,367	3,648	67	2,254	1,394	5 years
Novak et al. [16]	2020	Sweden	512	125	70	41	84	12 years
Yu et al. [17]	2018	Taiwan	1,267	306	N/A	N/A	N/A	10 years
Lofvenmark et al. [18]	2009	Sweden	149	29	76	10	19	1 year
Reeves et al. [19]	2014	Canada	10,048	2,288	72.24	882	1,406	1 year
Kraav et al. [20]	2020	Finland	2,682	2,682	51.8	2,682	N/A	23 years
Kitamura et al. [21]	2013	Japan	5,845	1,075	69	717	358	6 years
Benito et al. [22]	2024	Spain	298	115	75.8	52	63	362 days
Saito et al. [23]	2024	Japan	1,637	795	81	N/A	N/A	1 year
Blakoe et al. [24]	2021	Denmark	7,169	749	65.4	485	264	1 year
Case et al. [25]	1992	USA	1,234	202	61	71	131	4 years

The mean age was absent in one study [17], one study included only male participants [20], and the number of genders living alone was lacking in two studies [17,23]. The largest sample size was 32,367 [15], and 149 was the smallest sample size [13]. The study design included in this review is cohort (13-23), a cross-sectional study [24], and a randomized control trial [25]. The commonly reported CVDs include coronary artery disease (CAD) [13-15,21,24,25], heart failure [18,22,23], and hypertension [19]. A study by Kraav et al. [20] has the largest sample size of those living alone, while Lofvenmark et al. [18] has the smallest number of those living alone.

Impact and Outcome

The included studies mentioned the following impact among individuals living with loneliness when compared with individuals not living alone: increased risk of mortality [13,14,16,17,20-24], increased risk of stenosed vessels [13], hostility [14], depression [16,17,20], increased risk of major adverse cardiovascular events (MACE) [15,21,25], increased hospitalization/readmission [18,19,22], increased number of days in the hospital [18,19], and late arrival to the emergency room at onset of symptoms [19].

The most common outcomes reported among individuals living with loneliness when compared with individuals not living alone in the included articles are all-cause mortality, cardiovascular death, recurring CAD, stroke, hospital admission, and heart failure. Other outcomes are shown in Table 2 along with the number of individuals affected. Novak et al. [16] reported an increase in mortality among women, Gandhi et al. [15] reported an increase in mortality among men, and Case et al. [25] reported an increased chance of cardiac events in divorced marriages.

**Table 2:** Outcome of loneliness in individuals living with cardiovascular disease CAD: Coronary artery disease

Outcome	Number of patients
All-cause mortality	2,968
Cardiovascular death	360
Recurrent CAD	637
Recurrent Stroke	2,410
Recurrent Heart failure	121
Hospital readmission	1,409
Recurrent Pulmonary congestion	42
Blood transfusion	9
Major bleeding	51
Major adverse cardiac events	376
Late arrival to the emergency department at onset of symptom	

The search strategy used to synthesize articles on various databases is shown in Table 3.

**Table 3:** Search strategy

Search Strategy	Database	Outcome
(Self-isolation) AND (cardiovascular diseases)	PubMed	1852
(Loneliness) AND (cardiovascular diseases)	PubMed	386
"Loneliness" "cardiovascular diseases"	Google Scholar	980
"Self-isolation" "cardiovascular diseases"	Google Scholar	980

Tables 4-6 display the JBI critical appraisal tool for the included articles for cohort, cross-sectional, and randomized control trials.

**Table 4:** JBI cohort critical appraisal table JBI: Joanna Briggs Institute

Checklist question	Ohama et al. [13]	Brummett et al. [14]	Gandhi et al. [15]	Novak et al. [16]	Yu et al. [17]	Lofvenmark et al. [18]	Reeves et al. [19]	kraav et al. [20]	Kitamura et al [21]	Benito et al [22]	Saito et al [23]
Were the two groups similar and recruited from the same population?	Y	Y	Y	Y	Y	Y	Y	Y	Y	Y	Y
Were the exposures measured similarly to assign people to both exposed and unexposed groups?	Y	Y	Y	Y	Y	Y	Y	Y	Y	Y	Y
Was the exposure measured in a valid and reliable way?	Y	Y	Y	Y	Y	Y	Y	Y	Y	Y	Y
Were confounding factors identified?	Y	TY	Y	Y	Y	Y	Y	Y	Y	Y	Y
Were strategies to deal with confounding factors stated?	Y	Y	Y	Y	Y	N	N	Y	Y	Y	Y
Were the groups/participants free of the outcome at the start of the study (or at the moment of exposure)?	Y	Y	Y	N	Y	N	Y	Y	N	Y	Y
Were the outcomes measured in a valid and reliable way?	Y	Y	Y	Y	Y	Y	Y	Y	Y	Y	Y
Was the follow-up time reported and sufficient to be long enough for outcomes to occur?	Y	Y	Y	Y	Y	Y	Y	Y	Y	Y	Y
Was follow up complete, and if not, were the reasons to loss to follow-up described and explored?	Y	Y	Y	Y	Y	Y	Y	Y	Y	Y	Y
Were strategies to address incomplete follow-up utilized?	N	N	N	N	N	N	N	N	N	N	N
Was appropriate statistical analysis used?	Y	Y	Y	Y	Y	Y	Y	Y	Y	Y	Y

**Table 5:** JBI cross-sectional critical appraisal tool JBI: Joanna Briggs Institute

Checklist question	Blakoe et al. [24]
Were the criteria for inclusion in the sample clearly defined?	Y
Were the study subjects and the setting described in detail?	Y
Was the exposure measured in a valid and reliable way?	Y
Were objective, standard criteria used for measurement of the condition?	Y
Were confounding factors identified?	Y
Were strategies to deal with confounding factors stated?	Y
Were the outcomes measured in a valid and reliable way?	Y
Was appropriate statistical analysis used?	Y

**Table 6:** JBI randomized control trial critical appraisal tool JBI: Joanna Briggs Institute

Checklist question	Case et al. [25]
Was true randomization used for assignment of participants to treatment groups?	Y
Was allocation to treatment groups concealed?	Y
Were treatment groups similar at the baseline?	Y
Were participants blind to treatment assignment?	Y
Were those delivering the treatment blind to treatment assignment?	Y
Were outcome assessors blind to treatment	Y
Were treatment groups treated identically other than the intervention of interest?	Y
Were outcomes measured in the same way for treatment groups?	Y
Were outcomes measured in a reliable way	Y
Was follow up complete and if not, were differences between groups in terms of their follow up adequately described and analyzed?	Y
Were participants analyzed in the groups to which they were randomized?	Y
Was appropriate statistical analysis used?	Y
Was the trial design appropriate and any deviations from the standard RCT design (individual randomization, parallel groups) accounted for in the conduct and analysis of the trial?	Y

Discussion

This systematic review analyzed 13 studies across eight countries, encompassing 63,978 participants, of whom 12,110 were identified as living alone or socially isolated. The findings provide compelling evidence that loneliness is associated with adverse cardiovascular disease (CVD) outcomes, including increased mortality, recurrent cardiovascular events, and higher hospitalization rates. These results align with existing literature and highlight the critical role of social determinants in CVD prognosis.

Loneliness and Mortality in Individuals With CVD

The present review identifies a significant association between loneliness and increased mortality in individuals with CVD, with 2,968 cases of all-cause mortality and 360 cases of cardiovascular-related death reported across included studies. This aligns with prior meta-analyses demonstrating that social isolation and loneliness independently increase the risk of cardiovascular and overall mortality. Valtorta et al. [26] reported a 29% increased risk of coronary heart disease and a 32% increased risk of stroke among socially isolated individuals. Similarly, Holt-Lunstad et al. [27] emphasized that the detrimental effects of social isolation on mortality are comparable to those of well-established cardiovascular risk factors such as smoking, obesity, and hypertension.

The biological mechanisms underlying this association have been widely studied. Loneliness has been linked to increased hypothalamic-pituitary-adrenal (HPA) axis activity, leading to elevated cortisol levels, heightened systemic inflammation, and autonomic dysregulation. These factors contribute to endothelial dysfunction, increased arterial stiffness, and a higher incidence of cardiovascular mortality [28]. The findings from the present review further support the notion that addressing social isolation should be considered an essential component of cardiovascular disease management.

Psychosocial Consequences: Depression and Hostility

Several studies included in this review reported psychological consequences of loneliness, including depression [29,30] and hostility [31]. These findings are consistent with existing research demonstrating that loneliness exacerbates mental health disorders, which in turn contribute to worse cardiovascular outcomes. Depression has been identified as a key risk factor for CVD due to its association with chronic inflammation, increased platelet aggregation, and poor adherence to medical therapy [32].

Cacioppo et al. [28] further highlighted that prolonged loneliness leads to increased sympathetic nervous system activation and dysregulation of the immune response, which may accelerate the progression of atherosclerosis. The presence of hostility, as observed in this review, has also been linked to heightened cardiovascular risk, as it is associated with increased catecholamine secretion, endothelial dysfunction, and greater susceptibility to hypertension [33].

Loneliness and Increased Risk of Major Adverse Cardiovascular Events (MACE)

A key finding of this review is the association between loneliness and an increased risk of major adverse cardiovascular events (MACE) [29,34]. These results align with previous research, such as Hakulinen et al. [35], who found that socially isolated individuals had a 43% higher risk of MACE than those with strong social networks. The mechanisms underlying this association may involve chronic inflammation, increased blood pressure variability, and suboptimal medication adherence among individuals experiencing social isolation [35,36].

The increased prevalence of coronary artery disease (CAD) among socially isolated individuals further supports these findings. Loneliness has been associated with increased vascular inflammation and higher rates of atherosclerotic plaque development, contributing to stenosed vessels and recurrent cardiovascular events [37].

Hospitalization, Delayed Emergency Response, and Readmission

Another critical finding is the increased risk of hospitalization and hospital readmission among socially isolated individuals. This review identified 1,409 cases of hospital readmission, an increased number of hospital days, and a tendency for late arrival at the emergency department during acute cardiovascular events. These results are supported by previous studies indicating that socially isolated individuals exhibit poor health-seeking behaviors, which contribute to delayed intervention and worse outcomes [38].

Moreover, Cohen-Mansfield et al. [39] reported that lonely patients experience longer hospital stays due to limited post-discharge support, increasing their risk of complications and mortality. The present findings highlight the urgent need for targeted interventions aimed at improving social support structures to enhance timely healthcare access and reduce hospital readmission rates among individuals with CVD.

Recurrent Cardiovascular Events: CAD and Stroke

The association between loneliness and recurrent cardiovascular events, particularly recurrent coronary artery disease (637 cases) and recurrent stroke (2,410 cases), was another significant finding of this review. Previous epidemiological studies, such as those by Steptoe et al. [36], have demonstrated that loneliness can double the risk of stroke and CAD recurrence due to increased inflammatory responses, poor medication adherence, and behavioral risk factors such as physical inactivity and smoking.

Furthermore, lonely individuals often experience higher levels of pro-inflammatory cytokines, including interleukin-6 (IL-6) and tumor necrosis factor-alpha (TNF-α), which contribute to endothelial dysfunction and increased thrombogenic potential [35,36]. These findings reinforce the importance of social interventions in secondary prevention strategies for cardiovascular disease.

Clinical Implications

The results of this review underscore the need for healthcare providers to incorporate social support assessments into routine cardiovascular risk evaluations. Given the strong evidence linking loneliness to increased morbidity and mortality in individuals with CVD, targeted interventions such as social prescribing, peer support programs, and community-based initiatives should be prioritized.

Furthermore, policymakers should consider integrating social determinants of health into CVD prevention and management strategies. Addressing loneliness through structured interventions, such as digital social connectivity programs and in-person social support groups, may help mitigate its negative impact on cardiovascular outcomes.

Strengths and Limitations

This systematic review offers several strengths that enhance the reliability and applicability of its findings. One of its primary strengths is the large and diverse sample size, which includes 63,978 participants from eight different countries. This geographical and cultural diversity improves the generalizability of the results, making them applicable across various populations and healthcare systems. Additionally, the inclusion of studies spanning from 1992 to 2024 allows for an extensive evaluation of the long-term impact of loneliness on CVD outcomes. The review also benefits from the incorporation of studies with substantial follow-up periods, ranging from just under one year to 23 years, which provides valuable insights into the long-term effects of social isolation on cardiovascular health.

Another notable strength of this review is its comprehensive assessment of a wide range of cardiovascular outcomes, including all-cause mortality, cardiovascular death, recurrent coronary artery disease, stroke, heart failure, and hospital readmission. By encompassing multiple cardiovascular conditions, the study provides a holistic perspective on the consequences of loneliness and social isolation in individuals living with CVD. Furthermore, the inclusion of various study designs, cohort studies, a cross-sectional study, and a randomized controlled trial, enhances the robustness of the findings by incorporating different methodological approaches. While cohort studies allow for a longitudinal examination of causality, the inclusion of an RCT provides a higher level of evidence regarding intervention-based outcomes.

Despite these strengths, several limitations should be acknowledged. One of the main challenges is the heterogeneity in study designs and methodologies. The included studies varied in their definitions and measurements of loneliness and social isolation, with some relying on self-reported surveys while others used indirect or objective measures. This variability makes direct comparisons between studies more difficult and may contribute to inconsistencies in findings. Additionally, loneliness and social isolation, while related, are distinct constructs: one being the subjective feeling of being alone and the other referring to an objective lack of social connections. The lack of a standardized approach to defining and measuring these factors may have influenced the results, leading to variations in reported associations with cardiovascular outcomes.

Furthermore, while the review establishes a strong association between loneliness and poor cardiovascular outcomes, the majority of included studies were observational in nature, limiting the ability to draw definitive causal conclusions. The absence of more intervention-based studies makes it difficult to determine whether reducing loneliness would directly improve cardiovascular health. Future research should focus on randomized trials evaluating targeted social interventions to assess their effectiveness in mitigating cardiovascular risk.

Finally, the exclusion of non-English and unpublished studies introduces the possibility of publication bias. Research conducted in non-English-speaking regions or studies with null findings may have been overlooked, potentially affecting the completeness of the review. Additionally, psychological mediators such as depression, anxiety, and chronic stress, factors that often accompany loneliness, were not consistently examined across studies, despite their potential influence on cardiovascular health. Future studies should further investigate the interplay between mental health, social isolation, and cardiovascular outcomes to provide a more nuanced understanding of the relationship.

Despite these limitations, this systematic review provides valuable insights into the impact of loneliness and social isolation on cardiovascular disease. The findings reinforce the importance of considering social determinants of health in cardiovascular risk assessments and highlight the need for targeted interventions to address loneliness in clinical practice.

## Conclusions

This systematic review provides strong evidence that loneliness and social isolation are significant risk factors for adverse cardiovascular outcomes, including increased mortality, recurrent cardiovascular events, and prolonged hospitalization. The findings highlight the importance of addressing social determinants of health in cardiovascular disease management. The evidence suggests that socially isolated individuals are at greater risk of delayed healthcare access, reduced post-hospitalization support, and increased psychological distress, further exacerbating their cardiovascular burden. However, peer support programs and community-based initiatives may help mitigate the adverse effects of loneliness on heart health.
